# Do thin, overweight and obese children have poorer development than their healthy-weight peers at the start of school? Findings from a South Australian data linkage study

**DOI:** 10.1016/j.ecresq.2015.10.007

**Published:** 2016-03-02

**Authors:** Anna Pearce, Daniel Scalzi, John Lynch, Lisa G. Smithers

**Affiliations:** a School of Public Health, University of Adelaide, Mail drop DX 650550, Adelaide 5005, Australia; b Population, Policy and Practice, UCL Institute of Child Health, 30 Guilford Street, London WC1N 1EH, United Kingdom; c School of Social & Community Medicine, University of Bristol, BS82BM, United Kingdom

**Keywords:** Australian Early Development Census, Early Development Instrument, Childhood thinness, Childhood overweight and obesity, Data linkage, Child development

## Abstract

Little is known about the holistic development of children who are not healthy-weight when they start school, despite one fifth of preschool-aged children in high income countries being overweight or obese. Further to this, there is a paucity of research examining low body mass index (BMI) in contemporary high-income populations, although evidence from the developing world demonstrates a range of negative consequences in childhood and beyond. We investigated the development of 4–6 year old children who were thin, healthy-weight, overweight, or obese (as defined by BMI z-scores) across the five domains of the Australian Early Development Census (AEDC): Physical Health and Wellbeing, Social Competence, Emotional Maturity, Language and Cognitive Skills, and Communication Skills and General Knowledge. We used a linked dataset of South Australian routinely collected data, which included the AEDC, school enrollment data, and perinatal records (*n* = 7533). We found that the risk of developmental vulnerability among children who were thin did not differ from healthy-weight children, after adjusting for a range of perinatal and socio-economic characteristics. On the whole, overweight children also had similar outcomes as their healthy-weight peers, though they may have better Language and Cognitive skills (adjusted Risk Ratio [aRR] = 0.73 [95% CI 0.50–1.05]). Obese children were more likely to be vulnerable on the Physical Health and Wellbeing (2.20 [1.69, 2.87]) and Social Competence (1.31 [0.94, 1.83]) domains, and to be vulnerable on one or more domains (1.45 [1.18, 1.78]). We conclude that children who are obese in the first year of school may already be exhibiting some developmental vulnerabilities (relative to their healthy-weight peers), lending further support for strategies to promote healthy development of preschoolers.

## Highlights

• Little is known about how child development varies by BMI at the start of school.
• Outcomes for thin and overweight children are similar as healthy-weight children.
• Obese children are more likely to be developmentally vulnerable at the start of school, compared to health weight children.
• Obese children have higher vulnerability with physical health and wellbeing, compared to healthy weight children.

## 1 Introduction

The transition into primary school is considered to be an important period in the life course. A child's ability to fully benefit from, and participate in, school life is dependent upon their physical, cognitive, and socio-emotional development (Janus et al., 2007; UNICEF & Britto, 2012). Every child has the right to be physically healthy, including being free from illness and possessing the fine and gross motor skills (such as the ability to hold a pencil and to move around independently) to allow them to engage in classroom activities. Other essential foundations for learning include cognitive abilities (such as knowledge of the alphabet, basic numeracy, and logic) and language skills (reading, speaking, and understanding). Socio-emotional behaviors including emotional regulation, attention, social relationships, and awareness, as well as attitudes (curiosity, persistence, creativity, and problem solving) are supportive of learning (Janus et al., 2007; UNICEF & Britto, 2012). These aspects of child development have been linked to later school achievement (Brinkman, Gregory, Harris, Hart, Blackmore, & Janus, 2013; Forget-Dubois et al., 2007; Oberle, Schonert-Reichl, Hertzman, & Zumbo, 2014) and subsequently to health, well-being, and social circumstances (such as employment status) in adulthood (Hertzman & Wiens, 1996; Law, 2009; Lynch & Smith, 2005).

There is recognition of the potential for early child development to improve health and well-being (Jolly, 2007), and supporting early child development is a priority of governments around the globe (Abbott et al., 2002; Allen, 2011; Australian Institute of Health and Welfare, 2011; Council of Australian Governments, 2009; Council of Ministers of Education, 2014; UNICEF, 2007). This has prompted the design of schemes such as the Australian Early Development Census (AEDC), which involves monitoring aspects of early child development that are relevant for understanding children's preparedness to learn at school and is indicative of later school performance (Brinkman et al., 2012; Janus et al., 2007).

All aspects of child development, including cognition, socio-emotional well-being, and motor skills, are dependent upon physical and nutritional well-being. The interdependence between different aspects of health and well-being (e.g., mental health and chronic disease) is increasingly acknowledged by researchers and policy makers alike (Australian Institute of Health and Welfare, 2012; Barnett et al., 2012; Department of Health and Department for Children Schools and Families, 2009). Yet in developed countries, little is known about the general development of children who are not of a healthy body mass index (BMI). This is despite dramatic increases in rates of overweight and obesity (Hardy et al., 2012; Wang et al., 2013); and a wealth of evidence from low- to middle-income countries on the detrimental impacts of impeded growth throughout infancy and childhood (Grantham-McGregor, Fernald, & Sethuraman, 1999a; Nyaradi, Li, Hickling, Foster, & Oddy, 2013).

### 1.1 Overweight, obesity and early childhood development

In recent decades, childhood overweight and obesity have increased dramatically in Australia (Booth, Wake, Armstrong, Chey, Hesketh, & Mathur, 2001; Hardy et al., 2012) and in other countries (Chinn & Rona, 2001; Ogden et al., 1994; Stamatakis, Primatesta, Chinn, Rona, & Falascheti, 2005; Wang et al., 2013), with some signs of levelling off (Hardy et al., 2012; Stamatakis, Wardle, & Cole, 2009). Overweight and obesity have been associated with poorer outcomes in later childhood, including reduced self-esteem and psychosocial well-being (Griffiths, Parsons, & Hill, 2010), and the development of cardiovascular risk factors and metabolic disorders (Lobstein, Baur, & Uauy, 2004). In adulthood, overweight and obesity have been linked to a range of negative outcomes, including cancer (Guh et al., 2009), cardiovascular disease (Guh et al., 2009), and reduced healthy life expectancy (Nagai et al., 2012; Steensma et al., 2013).

There is a paucity of research examining the association between overweight, obesity, and development in young children, and of the studies that are available, findings have been mixed. There is some evidence that obese children have poorer socio-emotional well-being and behavior (Cawley & Spiess, 2008; Drukker, Wojciechowski, Feron, Mengelers, & Van Os, 2009; Griffiths, Dezateux, & Hill, 2011; Sawyer et al., 2006), cognition and language (Cawley & Spiess, 2008; Kamijo et al., 2012), and academic scores (Cottrell, Northrup, & Wittberg, 2007). Obese children have also been shown to be at increased risk of asthma or wheezing (Wake et al., 2013; Wake, Hardy, Sawyer, & Carlin, 2008), poor scores on global measures of health (Wake et al., 2013), lower daily activity skills, and fine and gross motor abilities (Castetbon & Andreyeva, 2012; Cawley & Spiess, 2008; D’Hondt et al., 2013; Mond, Stich, Hay, Kraemer, & Baune, 2007). In many cases, these differences are small (Li, Dai, Jackson, & Zhang, 2008; Sawyer et al., 2006; Wake et al., 2013; Wake et al., 2008), and several studies show inconsistencies across outcomes, genders, or age groups (Jansen, Mensah, Clifford, Nicholson, & Wake, 2013; Jansen, Mensah, Clifford, & Tiemeier et al., 2013; Kamijo et al., 2012; Lawlor et al., 2005; Sawyer et al., 2006), or that the relationships are confounded by socio-economic circumstances (Datar, Sturm, & Magnabosco, 2004; Li et al., 2008). It has been postulated that null findings may be due to some studies examining overweight and obese children as one group (Griffiths et al., 2011). It is possible that any effect on child development may be more evident as the extent to which a child is overweight increases, and consequently, there is a need to examine overweight and obesity separately.

### 1.2 Thinness and Early Childhood Development

Recently, age- and gender-adjusted BMI cut-offs for thinness (low BMI) were created by Cole, Flegal, Nicholls, and Jackson (2007), to complement the International Obesity Taskforce (IOTF) cut-offs for childhood overweight and obesity (Cole, Bellizzi, Flegal, & Dietz, 2000). In high-income countries, much less attention has been paid to the determinants and consequences of childhood thinness than overweight and obesity, even though there is evidence that thinness remains a public health issue (Armstrong & Reilly, 2003; Boddy, Hackett, & Stratton, 2009; O'Dea & Amy, 2011; Wake et al., 2013). The majority of evidence refers to the impact of more chronic measures of impeded growth in early childhood (such as stunting) on development. Nevertheless, it is thought that moderate or mild degrees of thinness can impede development, including language, intelligence, attention, reasoning, and visuospatial functioning (Nyaradi, Li, Hickling, & Foster et al., 2013; Sandjaja et al., 2013). There is a dearth of research examining the association between thinness and child development in high-income countries, particularly in preschool children, and using measures of development that capture the preparedness of children to fully benefit from and participate in school life. The limited evidence base indicates that thinness is associated with worse academic scores (Cottrell et al., 2007), poorer global health (Wake et al., 2013; Wijga et al., 2010), higher special health care needs (e.g., having a chronic health condition; Wake et al., 2013), and possibly higher rates of infection and conditions which limit daily functioning (Wijga et al., 2010). On the other hand, studies have found that children who are thin are no different from healthy-weight children in terms of their behavior and socio-emotional well-being (Wake et al., 2013), susceptibility to respiratory infections, number of visits to general practitioners, school absenteeism due to illness (Wijga et al., 2010), and motor skills (Castetbon & Andreyeva, 2012). Indeed, one study found that thin children had a reduced risk of asthma (Wake et al., 2013), and another that thin children were less likely to display behavioral problems (Drukker et al., 2009), when compared with healthy-weight children. However, various definitions of thinness (or low BMI) were used in these studies, limiting comparability.

### 1.3 Nutrition and early child development

BMI is a widely acknowledged marker of malnutrition in population research (de Onis & Blössner, 2003). For example, thinness can occur when children do not have sufficient energy and protein (de Onis, Monteiro, Akré, & Glugston, 1993); and protein-energy malnutrition often goes hand-in-hand with other nutritional problems, such as deficiencies in micronutrients (Grantham-McGregor et al., 1999a). At the other end of the BMI spectrum, overweight and obesity reflect an excess of the energy needed for childhood growth and activity (de Onis & Blössner, 2003). Despite overconsumption of energy, obese individuals may still be lacking in some macro-nutrients (e.g., protein) and also micro-nutrients (such as iron) that are needed for healthy development (Burkhalter & Hillman, 2011; Tanumihardjo et al., 2007).

While our understanding of the relationship between nutrition and child development requires further advancement, there is some evidence that children who are deficient in macro-nutrients (such as protein) and micro-nutrients (such as iron and zinc), have poorer cognitive, behavioral, and motor development, as well as physical illness (Burkhalter & Hillman, 2011; Grantham-McGregor, Fernald, & Sethuraman, 1999b). For example, children who are iron deficient display higher rates of inhibition and clinginess to their caregiver (Bellisle, 2004; Grantham-McGregor et al., 1999b; Nyaradi, Li, Hickling, & Foster et al., 2013; Stevenson, 2006).

As some nutrients have been linked to child development, a number of studies have sought to examine the association between general dietary patterns and child development (Nyaradi, Li, Hickling, & Whitehouse et al., 2013; Smithers et al., 2012, Smithers et al., 2013), to allow for the fact that individuals consume combinations of foods and nutrients as part of an overall diet. These studies indicated that healthier dietary patterns (such as those which are rich in whole grains and vegetables, or that consist of home cooked food) may have small benefits to intelligence and cognition (Nyaradi, Li, Hickling, & Whitehouse et al., 2013; Nyaradi, Li, Hickling, & Foster et al., 2013; Smithers et al., 2012, Smithers et al., 2013). Attention has also been paid to the consumption of breakfast and whether it has benefits for cognition and behavior; intuitively, the consumption of breakfast after a period of overnight fasting should be beneficial for physical and mental well-being, although the majority of evidence only points toward benefits in adolescents or malnourished children (Hoyland, Dye, & Lawton, 2009; Stevenson, 2006). Nevertheless, children living in families that report that they sometimes or often do not get enough food to eat have been shown to have worse academic outcomes (Burkhalter & Hillman, 2011), and there are anecdotal reports of children coming to school hungry in high-income countries such as Australia, which, in turn, affects short-term concentration levels and the ability to learn (Australian Bureau of Statistics, 2014; Foodbank Australia, 2013).

### 1.4 The present study

The aim of this study was to investigate whether children who do not have a healthy BMI are more likely to be developmentally vulnerable on a global measure of child development at the start of school. We investigated five important developmental domains (Physical Health and Wellbeing, Social Competence, Emotional Maturity, Language and Cognitive Skills, and Communication Skills and General Knowledge), and examined categories of weight status spanning the full spectrum of BMI (thinness, healthy weight, overweight, and obesity), as we anticipated that the effects of low and high BMI, on different aspects of child development, would vary. We did this using four routinely collected government data sources in South Australia, which offer a unique opportunity to examine associations between BMI status and children's development, and adjust for a wide range of potential confounding variables relating to the child's demographic, socio-economic, and birth characteristics.

## 2 Method

The study sample comprised children who took part in the 2009 AEDC in the first year of school (mean age 5.2 years) and received a preschool health check (mean age 4.8 years) at which height and weight were collected. When possible, potential confounding factors were obtained from these two datasets. Additional potential confounding variables were obtained from two further datasets: perinatal hospital records and the student school enrollment census. The measures used in the analysis, and the datasets they were derived from, are now described.

### 2.1 Outcome: children’s developmental vulnerability

The census of child development, known as the AEDC, is conducted by the Australian federal government every three years (www.aedc.gov.au). Alongside questions regarding child demographics, and teacher and classroom characteristics, the AEDC includes a validated questionnaire (Janus, Brinkman, & Duku, 2011), which was adapted to the Australian context from the Canadian Early Development Instrument (EDI; Janus et al., 2007), and is designed to capture a holistic measure of child development. The AEDC has equivalent psychometric properties compared to the Canada and United States EDI measures (Janus et al., 2011; Janus & Offord, 2007), demonstrating good content, construct, and predictive validity (Brinkman & Blackmore, 2003; Brinkman et al., 2007), and excellent internal reliability (i.e., internal consistency; Janus et al., 2011; Janus & Offord, 2007).

**Fig. 1:**
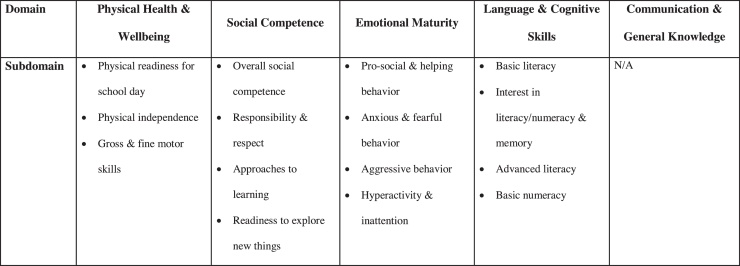
Domains and subdomains of development captured in the Australian Early Child Development Census (AEDC).

The questionnaire is filled in by school teachers for children attending their first year of school (usually in the second school term; Centre for Community Child Health and Telethon Institute for Child Health Research, 2011). It is made up of 95 questions, which are used to create scores (ranging from 0 to 10) across five developmental domains: Physical Health and Wellbeing, Social Competence, Emotional Maturity, Language and Cognitive Skills, and Communication Skills and General Knowledge. Each of the domains (apart from Communication Skills and General Knowledge) is made up of several subdomains (see Fig. 1). The scores from each of the domains are adjusted for age (in years; The Social Research Centre Pty., Ltd, 2014) and, according to national reporting practices, children with scores below the 10th percentile on each domain are categorized as being developmentally vulnerable. An additional global measure of child development is also created, which captures whether a child is vulnerable on one or more of the domains. All domains of the AEDC, and the global measure, demonstrate internal consistency and predictive validity for later school achievement in Australia (Brinkman et al., 2013, Brinkman et al., 2007). In this analysis, we used data from the 2009 AEDC. We examined vulnerability on each of the five domains, and also the global measure of developmental vulnerability.

### 2.2 Exposure: BMI status

In South Australia, a preschool health check is freely available to all children prior to entering school (provided by the Child and Family Health Service, the Women's and Children's Health Network, South Australian Department of Health). Children's height, weight, hearing, vision, and oral health are assessed by community health nurses, at local health clinics or the child's preschool. We used children's height and weight data to estimate BMI (weight (kg)/height (m)^2^). BMIs were transformed to sex- and age-specific z-scores using the World Health Organization's (WHO) reference data for child growth (de Onis & World Health Organization, 2006; World Health Organization and Nutrition for Health, 2007) and the zanthro program for Stata. In accordance with the WHO reference data, children with height, weight or BMIs that were more extreme than five standard deviations (*SD*) above or below the mean were considered implausible and not included in the analysis. Children were categorized as thin, healthy, overweight, or obese using the International Obesity Taskforce age- and sex-specific cut-offs (Cole et al., 2000, Cole et al., 2007).

### 2.3 Confounding variables: socio-economic characteristics and factors related to birth

Potential confounding factors were selected *a priori* based on a causal model of children's weight status and child development, using directed acyclic graphs (visual representations of the temporal associations between a set of variables; Glymour, 2006). Variables representing common causes of the exposure (BMI status) and outcome (child development) were used to account for potential confounding. Where possible, confounding variables were obtained from the preschool health check and AEDC databases; additional variables were drawn from the perinatal and student school enrollment census databases, as now outlined.

Indigenous status (Aboriginal and/or Torres Strait Islander status: yes/no) of the child was obtained from the AEDC, as was area disadvantage (in quintiles, based on the Socioeconomic Index for Areas; Australian Bureau of Statistics, 2008). Remote/non-remote area of residence was examined using the Australian Remoteness Index for Areas (ARIA; Australian Institute of Health and Welfare, 2004), which was obtained from the preschool health check.

A number of characteristics relating to the child and their birth were obtained from perinatal hospital records: gender, maternal age at child's birth, maternal smoking during second half of pregnancy (yes/no), plurality (singleton/multiple birth), gestational age at birth (continuous), birth-weight-for-gestational age z-score (calculated using recently-published norms for births in Australia (Dobbins, Sullivan, Roberts, & Simpson, 2012)), and whether the mother experienced any complications during the perinatal period (including pre-pregnancy and gestational hypertension (normal/high) and diabetes (no diabetes/diabetes), which were defined using standard criteria (McLean, Scott, Keane, Sage, & Chan, 2001; Scott & Chan, 2012). Collection of these data by midwives and neonatal nurses using a Supplementary Birth Record (SBR) form and a companion guide are mandatory for all live births in South Australia. They are collated by the Perinatal Outcomes Unit, South Australian Department of Health, and have been validated against an audit of medical records (McLean et al., 2001).

A number of additional socio-economic variables were obtained from the school enrollment census (which contains enrollment information for all children attending government schools in South Australia): parental education (categorized as <year 12, year 12, diploma or above), parental employment (both parents employed, at least one employed, neither employed, or not stated/unknown), and eligibility for a school card (a scheme that offers financial assistance for educational expenses to low-income families; McLean et al., 2001). This information was derived from a form which must be completed by the children's parents/guardians at the start of school and again if the child changes school. Schools are expected to perform validation checks and report data to the state government Department for Education and Child Development annually (Department of Education and Children's Services, 2009). In 2009, 64% of children attended government schools in South Australia and 36% attended private (non-government schools) or were home schooled (Australian Bureau of Statistics, 2014).

### 2.4 Data linkage

Data from the four government datasets were linked by an independent agency to maintain confidentiality (SA-NT DataLink, 2014). Data custodians from the government departments provided basic identifiers such as name, age, gender, and address, to the data linkage agency, who then used a probabilistic linkage algorithm to match records from different datasets. To minimize mismatches of individuals across the datasets, the data linkage agency undertook a set of quality assurance checks and clerical review. As unique identification numbers (i.e., for health care cards) are not used in Australia, linkages are necessarily probabilistic and are based on key demographic information and therefore a small degree of error is to be expected. Linkage errors can occur from missed links or incorrect positive links (Guiver, 2011). Calculation of false linkages has not yet been undertaken in South Australia; however, Western Australia and New South Wales use similar systems and estimate false positive linkage errors of approximately 0.1% and 0.3% (Centre for Health Record Linkage, 2014; Holman, Bass, Rouse, & Hobbs, 1999), respectively. In less than 0.5% of cases, the information in the datasets did not uniquely identify each case, resulting in a very small number of duplicates (*n* = 27 in the child health check dataset and *n* = 142 in the AEDC). All duplicates were omitted prior to analysis.

## 2.5 Ethical approval

Approval for this study was given by the ethics committees of the South Australian Department of Health (377/06/2013) and the University of Adelaide (H-185-2011). Approval was also provided by the data custodians, who are representatives from the government departments that are responsible for the datasets. This study involved the use of de-identified data only.

## 2.6 Statistical analysis

Regression models were used to estimate associations between BMI category and vulnerability on each of the developmental domains, using binary regression to estimate the relative risk (RR) and 95% confidence interval (CI) before and after adjustment for confounding variables. All analyses were carried out in Stata SE 13 (TX, U.S.).

### 2.7 Analytic sample

The measures of child development were taken from the 2009 AEDC. In 2009, 93% of South Australian students had an AEDC checklist completed. In our dataset, the majority of children were aged 4–6 years when the AEDC was collected (99.9%, age mean = 5.1 years; median = 5 years). Children outside this age range were excluded from the current study (<0.1%), leaving *n* = 16,515 children.

Eighteen thousand, one hundred and forty children, who we estimated to be eligible for the 2009 AEDC (i.e., born in 2003 or 2004), had taken part in the preschool health check and had valid BMI data; of these 7533 took part in the AEDC (and 99.5% had data for all five domains). Fifty seven percent (*n *= 4323) of children were missing data on at least one of the confounding variables, with the highest levels of missing for variables collected in the school enrollment census (for example 51.3% [*n *= 3685] children did not have information on parental employment status). Children with complete information on all relevant variables (42.6%, *n *= 3210) tended to be more advantaged than the response sample (Table A1, online supplement). To minimize bias, missing data on the confounding variables (and remaining AEDC domains) were imputed, using multiple imputation by chained equations. Twenty datasets were generated, and results were combined using Rubin's rules (Little & Rubin, 2002). Imputation was carried out under a Missing At Random assumption (that the missing values could be explained by the observed data). The imputation model included all outcome, exposure and confounding variables. The characteristics of the imputed data were comparable with the response sample (Table A1, online supplement). Results are reported for the imputed sample, unless otherwise stated.

## 3 Results

Fifteen percent (*n* = 1100) of children were overweight, 6% were thin (*n* = 416) and 5% (*n* = 358) obese. A description of these children, in terms of socio-economic, demographic, and birth characteristics, is given in Table 1.

Table 2 shows the prevalence of developmental vulnerability for thin, healthy, overweight, and obese children. The prevalence of vulnerability among healthy-weight children ranged from 4.1% for Language and Cognitive Skills to 8.9% for Emotional Maturity; 18.5% were vulnerable on one or more of the domains.

Table 3 presents risk ratios (RR) (95% confidence intervals [CIs]) for vulnerability of each of the developmental domains, and vulnerability on one or more domains among thin, overweight, and obese children, compared with healthy-weight children. The unadjusted point estimate for obese children reflected a higher relative risk of developmental vulnerability on the majority of the domains. For example, they were more likely to be developmentally vulnerable in terms of Physical Health and Wellbeing (RR = 2.47 [95% CI: 1.91, 3.19]) and Social Competence (1.42 [1.03, 1.96]); they were also more likely to be vulnerable on one or more domains (1.60 [1.31, 1.96]). Few differences in development were observed between thin and healthy-weight children, whereas children who were overweight tended to have lower risks on developmental vulnerability, particularly for Language and Cognitive skills (0.73 [0.51, 1.05]). After adjusting for confounding factors, obese children were still twice as likely to be developmentally vulnerable on the Physical Health and Wellbeing domain (2.20 [1.69, 2.87]). RRs for Social Competence, and for vulnerability on one or more domains, were attenuated but persisted, particularly for the latter (1.45 [1.18, 1.78]). As in the unadjusted analyses, there were no observable differences between thin and healthy-weight children. The lower risks of developmental vulnerability in overweight children (compared to healthy-weight) were attenuated in many cases but persisted for Language and Cognitive skills (0.73 [0.50, 1.05]). The associations in the complete-case sample were similar (Table A2, online supplement). Due to the large differences in physical development seen across the BMI categories, we carried out additional exploratory analyses using the physical subdomains of the AEDC (data not shown). After adjusting for confounding factors, elevated risks were seen for Physical Independence (2.42 [1.87, 3.12]) and Gross and Fine Motor skills (1.97 [1.42, 2.74]), but less so for Physical Readiness for the School Day (1.26 [0.90, 1.75]) (which captures characteristics more closely related to social disadvantage than the other two subdomains, for example being over or under-dressed or coming to school hungry). As seen with the overall physical domain, no differences were observed for thin or overweight children.

## 4 Discussion

### 4.1 Summary of findings

Information linked across a number of routine datasets was employed to examine the association between BMI and child development, in over 7500 South Australian children at school entry. We applied Cole et al. (2007) cut-offs to differentiate between thinness and healthy weight, unlike many previous studies which combined these groups as the baseline. We found no discernible differences in the physical, social, emotional, cognitive, and communication development of thin children as compared to their healthy-weight peers. In addition, our findings indicate that only obese (and not overweight) children, at mean age 4.8 years, are more developmentally vulnerable a few months later. Specifically, obese children were approximately 30% more likely to be vulnerable in terms of social competence, although this was attenuated after adjustment. In addition, obese children were more than twice as likely to be developmentally vulnerable on the physical domain compared to healthy-weight children. This association remained after adjusting for a wide range of covariables, and across two physical subdomains (physical independence, and gross and fine motor skills).

### 4.2 Comparison with other findings

To our knowledge, our study is the first to examine the effects of thinness, overweight, and obesity (separately) on a global measure of child development that takes into account the physical, social, emotional, and cognitive development of children. If these effects are causal, we have shown that obesity appears to have a detrimental effect on some aspects of child development, particularly physical development, and to some extent social competence. In contrast, being overweight tended to have no effect on physical and social domains, yet possibly protective effects on the Language & Cognitive skills domain. We found that thin children appear to be no more or less likely to be developmentally vulnerable than their healthy-weight peers.

Of the studies that combined overweight and obese children together, some found elevated risks of poor mental health, social functioning, and physical health (Jansen, Mensah, Clifford, & Nicholson et al., 2013; Jansen, Mensah, Clifford, & Tiemeier et al., 2013); another found no relationship (Lawlor et al., 2005), although arguably, this study investigated children who were born before the onset of the obesity epidemic. It is possible that studies examining overweight (including obesity) may have seen bigger effect sizes for the obese children had they separated out these two groups. The few studies that have examined overweight and obesity separately, like our study, found that obesity had a greater detrimental impact on socio-emotional behavior (Griffiths et al., 2011) and physical health (Wake et al., 2013; Wijga et al., 2010).

We found that obese children were around 30% more likely to be vulnerable on the Social Competence domain, which refers to overall social competence, responsibility and respect, approaches to learning, and readiness to explore new things. Comparability of the AEDC domains with the developmental measures used in other studies is limited, though earlier findings have shown that children who are obese have poorer socio-emotional well-being and behavior (Griffiths et al., 2011; Wake et al., 2008), as measured on the Strengths and Difficulties Questionnaire (SDQ). Although the SDQ also captures emotional problems, in this study we found no association between BMI status and emotional maturity.

Obese children were more than twice as likely to be vulnerable on the Physical Health and Wellbeing domain. A body of research has found that obese children have greater special health care needs and are more likely to experience wheeze and possibly infections and health-related limitations (Wake et al., 2013; Wijga et al., 2010). While the Physical Health and Wellbeing domain of the AEDC does not capture specific health conditions in this way, it is designed to encapsulate any barriers that might impede a child's preparedness to learn and participate in school life. It is therefore possible that the presence of health problems, such as wheezing, in a child might be reflected in teachers' ratings on the AEDC.

The information collected in the AEDC not only allowed us to examine five broad developmental domains, but also to investigate more specific aspects of development using the subdomains. We did this for the Physical Health and Wellbeing domain because its association with obesity was especially high; (obese children were more than twice as likely to have poor physical development). We found that this elevated risk remained in two of the three sub-domains (Physical Independence, and Gross and Fine motor skills). Several studies have found that overweight (including obese) children have poorer gross motor skills than healthy-weight children (Castetbon & Andreyeva, 2012; Mond et al., 2007; Morano, Colella, & Caroli, 2011), though associations tended to be for skills related directly to their weight (such as jumping and hopping), whereas fine motor skills or general coordination were not affected (Castetbon & Andreyeva, 2012).

We found that vulnerability on the Language and Cognitive Skills domain did not vary between healthy-weight and obese children, unlike two studies from America and Europe, which found a detrimental effect on cognitive development and academic scores (Cawley & Spiess, 2008; Cottrell et al., 2007). However, one of these studies concentrated on older children (Cottrell et al., 2007) and it is possible that the impacts of BMI on cognition and academic performance is cumulative or become apparent at later ages when cognitive abilities have undergone further development. There was also the suggestion that overweight children had better language and communication skills; to our knowledge no other study has shown this finding and this should be examined further.

### 4.3 Strengths and limitations

This is the first study to explore the association between BMI and a holistic measure of early child development (the AEDC). Using IOTF cut-offs for overweight and obesity (Cole et al., 2000, Cole et al., 2007), and cut-offs for thinness, we examined the full spectrum of BMI categories. We differentiated between overweight and obesity, because it has been postulated that null findings in earlier studies are the result of combining these groups. Furthermore, unlike many earlier studies, we separated out thinness from healthy-weight children, which allowed us to examine whether there was an increased risk to development associated with under-nutrition among children from an otherwise well-nourished, high-income country. The advantage of using a holistic measure of development such as the AEDC is that we were able to explore whether some aspects of child development were more strongly related to BMI than others.

Through linking to other routine datasets, such as perinatal hospital records and the school enrollment census, we were able to adjust for a wider range of covariables than typically used in analyses of routine data, and even some surveys. Nevertheless it is possible that the association we observed between obesity and child development is the result of unmeasured or residual confounding. For example, we were not able to adjust for family income, or attendance at childcare, both of which may be associated with child development (Brinkman et al., 2012; Onise, Lynch, Sawyer, & McDermott, 2010; Gialamas, Mittinty, Sawyer, Zubrick, & Lynch, 2014; Zoritch, Roberts, & Oakley, 2000) and with childhood overweight (Brown, Broom, Nicholson, & Bittman, 2010; D’Onise et al., 2010; Wang, Patterson, & Hills, 2002).

BMI status reflects whether the child's diet has been meeting their nutritional needs for an extended period of time. BMI is easier to collect and analyze, and subject to less measurement error than techniques for measuring the intakes of individual nutrients, such as a 24-h dietary recall. BMI is therefore a well-established marker of nutrition in research using population samples (de Onis & Blössner, 2003). That said, we acknowledge that, at an individual level, an unhealthy BMI will not always be the result of malnutrition. For example a low BMI could result from a period of illness (de Onis & Blössner, 2003). Equally, Cole's international BMI cut-offs provide a good measure of adiposity for monitoring weight at the population level; nevertheless they cannot provide an accurate measure of fat mass (or lean mass) in individuals. Since only a small proportion of children were severely thin in South Australia, we were unable to examine the relationship between child development and different grades of thinness. Therefore, it remains possible that children of very low BMI have worse developmental outcomes and future research should examine this.

All of our analyses were limited to children who had both BMI and AEDC data. We only had access to children's height and weight if they were recorded in the Women's and Children's Health Clinic database (as part of a routine preschool health check in a clinic or at preschool). Some children may have had their weight and height measured in alternative settings such as well-child checks conducted by General Practitioners (GP). Whether the children who attend the Women's and Children's Health Clinics differ from children who attend well-child checks by GPs is not known because no data are available for comparison. However, we hypothesize that children measured in GP clinics may be more likely to have higher levels of health need (for example a GP may decide to carry out a health check of a child with a chronic condition), or use private health care (and so have the check with a private GP rather than with a community health nurse). It is therefore possible that the data under-represent the unhealthiest children, or the children in the extremes of advantage and disadvantage. Children's height and weight were measured by community health nurses, and the AEDC was collected by school teachers, both of whom are likely to have reduced recall or social desirability biases, compared with parent report. While this is a strength of our data, it remains possible that teachers' perceptions of a child's weight status may bias their reporting of the AEDC items (for example, it is possible that teachers may be more attuned to the motor abilities of obese children than those who are healthy weight).

In the present analysis, we examined the association between BMI and a measure of child development captured at the start of school; future research should examine whether the association between pre-school BMI status and child development persists or changes into later childhood. Finally, we were unable to explore common underlying influences such as diet, chronic disease (for example cystic fibrosis), and rare genetic conditions (such as Prader-Willi syndrome) which may lead to extreme BMIs and be linked to impeded cognitive development, behavioral problems, and poorer physical well-being.

### 4.4 Implications for policy, practice and further research

Every child has the right to healthy development and the opportunity to fulfill their potential (United Nations General Assembly, 1989), but unfortunately, some groups of children fare worse than others. Inequalities in child development exist across countries (Grantham-McGregor et al., 2007) and also within them, regardless of national wealth (Australian Government, 2013; Bradbury, Corak, Waldfogel, & Washbrook, 2010; Brinkman et al., 2012; Hertzman, 2009; Hertzman et al., 2010). In this study, we have shown that obese children are more likely to be developmentally vulnerable than their healthy-weight peers even as they start school. In particular, they are less likely to have the physical attributes required to maximize their potential to benefit from schooling. These include fine and gross motor skills (including the ability to hold a pencil or climb stairs), and physical independence (for example being independent in toileting needs). These differences persisted after adjusting for a range of covariables, including a number of socio-economic characteristics. Our findings point to the detrimental impacts of obesity and not overweight on child development. Despite this, we stress the importance of focusing on overweight as well obesity, since overweight children are at a greater risk of becoming obese (Power, Lake, & Cole, 1997).

To our knowledge, this is the first study to examine the links between BMI status and a global measure of early child development. Furthermore it demonstrates the value of data linkage for enhancing routine data like the AEDC. Our analyses refer to 4–5 year old children, who had been attending primary school in Australia for approximately four months. Therefore, the associations observed cannot be arising as a result of school influences, but more likely from characteristics of parents, the home environment, childcare, and neighborhoods. While we know that early life influences, such as maternal pre-pregnancy BMI and sedentary behaviors are likely to be contributing to early childhood obesity (Hawkins, Cole, & Law, 2008; Hawkins & Law, 2006; Reilly et al., 2005), the majority of intervention studies for obesity prevention have focused on older children and the evidence base for preschool children is limited (D’Onise et al., 2010; Hesketh & Campbell, 2010; Waters et al., 2011). Obesity prevention trials implemented from birth have resulted in negligible effects (Campbell et al., 2013; Daniels et al., 2014; Wen et al., 2012), and there is insufficient evidence around the wider societal and policy influences, such as parental employment and childcare, on preschool obesity. Efforts to increase the evidence base around the prevention of childhood obesity before children start school, including intervention studies and causal analysis of secondary data, should be continued.

## 5 Conclusion

Around one fifth of children are overweight or obese by the time they start school. Every child has the right to healthy development, yet obese children are more likely to be developmentally vulnerable (especially with regard to social competence, and physical health and well-being). The potential benefits of obesity reduction for physical health conditions in adulthood (Guh et al., 2009; Lobstein et al., 2004) and life expectancy (Nagai et al., 2012; Steensma et al., 2013) have already been widely documented. The findings from this study imply that tackling early childhood obesity may also have positive impacts for child development, leading to improvements in academic achievement and ultimately a fairer and more economically productive society.

## Figures and Tables

**Table 1:** Characteristics of children according to their weight status in the imputed sample (*n* = 7553).

	Thin 6% (*n *= 416)	Healthy 75% (*n *= 5659)	Overweight 15% (*n* = 1100)	Obese 5% (*n *= 358)
Maternal age (years)^a^	29.6 ± 0.26	29.5 ± 0.07	29.6 ± 0.17	29.5 ± 0.3
Maternal smoking during pregnancy^b^	14%	16%	18%	24%
High blood pressure during pregnancy^b^	8%	8%	9%	10%
Diabetes during pregnancy^b^	3%	3%	3%	5%
Twins^b^	2%	2%	1%	1%
School card^b^	22%	18%	20%	28%

SEIFA^b^				
Quintile 1	19%	17%	21%	22%
2	20%	21%	21%	24%
3	18%	21%	25%	22%
4	23%	21%	16%	17%
Quintile 5	20%	20%	17%	15%
Lives in a remote area^b^	4%	4%	5%	2%
Male^b^	50%	53%	46%	51%
Child is Aboriginal or Torres Strait Islander^b^	3%	2%	2%	3%
Gestational age at birth (wk)^a^	38.9 ± 0.11	39.0 ± 0.02	39.2 ± 0.47	39.1 ± 0.08
Birth weight (z-score)^a^	−0.49 ± 0.05	−0.19 ± 0.01	0.31 ± 0.03	0.39 ± 0.06
No employed parent in household^b^	3%	2%	3%	2%
No parent completed year 12^b^	20%	21%	22%	31%

Percentages may not sum to 100% due to rounding.

a values are mean ± SE.

b values are%. SEIFA: socio-economic indexes for areas.

**TABLE 2:** Proportion of children within each weight category considered vulnerable on each domain of the Australian Early Child Development Census (AEDC) (*n* = 7553).

	Physical health & wellbeing	Social competence	Emotional maturity	Language & cognitive skills	Communication & general knowledge	Vulnerable on one or more domains
Thin (*n* = 416)	8.7%	8.4%	9.4%	4.1%	6.7%	19.7%
Healthy-weight (*n* = 5659)	7.6%	7.9%	8.9%	4.1%	5.6%	18.5%
Overweight (*n *= 1100)	6.8%	7.0%	7.3%	3.0%	5.5%	16.1%
Obese (*n *= 358)	18.7%	11.2%	10.3%	5.3%	7.5%	29.6%

**Table 3:** Relative risk (95% confidence interval) of developmental vulnerability on the Australian Early Child Development Census (AEDC) according to weight category (healthy weight is the reference category)^a^.

	Physical health & wellbeing *RR* (95% CI)	Social competence *RR* (95% CI)	Emotional maturity *RR* (95% CI)	Language & cognitive skills *RR* (95% CI)	Communication & general knowledge *RR* (95% CI)	Vulnerable on one or more domains *RR* (95% CI)
Unadjusted in imputed sample, *N* = 7533						
Thin	1.14 (0.81, 1.60)	1.07 (0.76, 1.51)	1.05 (0.76, 1.46)	0.99 (0.61, 1.63)	1.19 (0.81, 1.76)	1.07 (0.85, 1.34)
Overweight	0.90 (0.70, 1.15)	0.89 (0.70, 1.13)	0.82 (0.64, 1.03)	0.73 (0.51, 1.05)	0.97 (0.73, 1.28)	0.87 (0.74, 1.02)
Obese	2.47 (1.91, 3.19)	1.42 (1.03, 1.96)	1.16 (0.83, 1.62)	1.29 (0.81, 2.06)	1.34 (0.90, 1.98)	1.60 (1.31, 1.96)

Adjusted^b^ in imputed sample, *N* = 7533						
Thin	1.11 (0.79, 1.57)	1.00 (0.71, 1.42)	1.01 (0.72, 1.40)	0.93 (0.56, 1.54)	1.13 (0.76, 1.68)	1.04 (0.83, 1.31)
Overweight	0.93 (0.72, 1.19)	0.95 (0.74, 1.21)	0.89 (0.70, 1.13)	0.73 (0.50, 1.05)	1.00 (0.75, 1.32)	0.89 (0.76, 1.05)
Obese	2.20 (1.69, 2.87)	1.31 (0.94, 1.83)	1.10 (0.78, 1.54)	1.06 (0.66, 1.71)	1.16 (0.78, 1.74)	1.45 (1.18, 1.78)

a Weight status was determined using z-scores of body mass index for age and International Obesity Taskforce cut-points (Cole et al., 2000, Cole et al., 2007). The reference category for the calculation of relative risks is healthy weight.

b Models were adjusted for the following potential confounders: maternal age, smoking during pregnancy, hypertension during pregnancy, diabetes during pregnancy, singleton birth, sex, gestational age at birth, birthweight for gestational age z-score, child Aboriginal and/or Torres Strait Islander status, parental education, parental occupation, school card, level of socioeconomic disadvantage of residence, and living in a remote area.
