# Supplementary material for: Do thin, overweight and obese children have poorer development than their healthy-weight peers at the start of school? Findings from a South Australian data linkage study
Source: Early Child Res Q. 2016 Mar 2;35:85–94. doi: 10.1016/j.ecresq.2015.10.007 (PMC4850238; doi:10.1016/j.ecresq.2015.10.007)
Supplement: Supplementary file 1 [file mmc1.docx]

ONLINE SUPLEMENTARY MATERIAL

TABLE A1

*Characteristics of the response, complete case and imputed samples*

|  | Response sample | Complete case n=3210 | Imputed  n=7533 |
| --- | --- | --- | --- |
| Maternal age (years)^a^ | 29.5 (0.06) | 29.5 | 29.5 |
| Maternal smoking during pregnancy^b^ | 16.7% (1199) | 15.6% | 16.7% |
| High blood pressure during pregnancy^b^ | 8.3% (602) | 8.7% | 8.3% |
| Diabetes during pregnancy^b^ | 3.2% (231) | 3.2% | 3.2% |
| Twins^b^ | 1.7% (125) | 1.7% | 1.7% |
| School card^b^ | 20.4% (999) | 12.0% | 19.3% |
| SEIFA^b^ |  |  |  |
| Quintile 1 | 18.2% (1368) | 14.6% | 18.2% |
| 2 | 20.9% (1576) | 20.6% | 20.9% |
| 3 | 21.4% (1608) | 22.7% | 21.4% |
| 4 | 20.2% (1522) | 20.1% | 20.2% |
| Quintile 5 | 19.3% (1451) | 22.0% | 19.3% |
| Lives in a remote area^b^ | 3.9% (294) | 5.2% | 3.9% |
| Male^b^ | 49.0% (3569) | 48.4 | 49.0% |
| Aboriginal and/or Torres Strait Islander^b^ Islanderb | 2.0% (152) | 1.4% | 2.0% |
| Gestational age at birth (wk)^a^ | 39.0 (0.02) | 39.1 | 39.0 |
| Birth weight (z-score) ^a^ | 0.02 (0.01) | 0.05 | 0.02 |
| No employed parent in household^b^ | 1.4% (51) | 1.4% | 2.2% |
| No parent completed year 12^b^ | 21.1% (818) | 20.7% | 21.8% |

Percentages may not sum to 100% due to rounding.

^a^ values are mean ± SE. ^b^values are %. SEIFA: Socio-Economic Indexes for Areas

TABLE A2

*Relative Risk (95% confidence intervals) of developmental vulnerability on the Australian Early Child Development Census (AEDC) according to weight category (healthy weight is the reference category), in complete sample (n ranges from 3202 to 3210, depending on AEDC domain)*

|  | Physical Health & Wellbeing  *RR* (95% CI) | Social Competence  *RR* (95% CI) | Emotional Maturity  *RR* (95% CI) | Language and Cognitive skills  *RR* (95% CI) | Communication & General Knowledge  *RR* (95% CI) | Vulnerable on one or more domains  *RR* (95% CI) |
| --- | --- | --- | --- | --- | --- | --- |
| *Unadjusted* | | | | | | |
| Thin | 0.84 (0.46, 1.58) | 1.32 (0.81, 2.16) | 0.70 (0.37, 1.26) | 0.74 (0.27, 1.99) | 0.71 (0.32, 1.60) | 0.92 (0.65, 1.31) |
| Overweight | 1.08 (0.75, 1.57) | 0.78 (0.51, 1.20) | 0.75 (0.51, 1.11) | 0.68 (0.34, 1.35) | 0.93 (0.58, 1.50) | 0.81 (0.63, 1.04) |
| Obese | 2.50 (1.67, 3.73) | 1.70 (1.05, 2.75) | 0.87 (0.47, 1.61) | 1.65 (0.78, 3.51) | 1.52 (0.82, 2.84) | 1.55 (1.16, 2.07) |
| *Adjusted*^b^ | | | | | | |
| Thin | 0.89 (0.49, 1.62) | 1.38 (0.85, 2.26) | 0.69 (0.38, 1.25) | 0.80 (0.30, 1.25) | 0.71 (0.33, 1.55) | 0.96 (0.68, 1.34) |
| Overweight | 1.11 (0.76, 1.61) | 0.81 (0.53, 1.22) | 0.77 (0.53, 1.13) | 0.64 (0.32, 1.27) | 1.00 (0.62, 1.60) | 0.82 (0.64, 1.04) |
| Obese | 2.29 (1.53, 3.42) | 1.61 (0.99, 2.61) | 0.86 (0.49, 1.52) | 1.27 (0.58, 2.77) | 1.48 (0.78, 2.81) | 1.45 (1.09, 1.94) |

^a^ Weight status was determined using z-scores of body mass index for age and International Obesity Taskforce cut-points ([Cole, et al., 2000](#_ENREF_32); [Cole, et al., 2007](#_ENREF_33)). The reference category for the calculation of relative risks is healthy weight. ^b^Models were adjusted for the following potential confounders; maternal age, smoking during pregnancy, hypertension during pregnancy, diabetes during pregnancy, singleton birth, sex, gestational age at birth, birthweight for gestational age z-score, Aboriginal and/or Torres Strait Islander status, parental education, parental occupation, school card, level of socioeconomic disadvantage of residence, and living in a remote area.
